# Editorial: Internet use and psychological well-being among children and adolescents

**DOI:** 10.3389/fpsyt.2023.1349082

**Published:** 2024-01-08

**Authors:** Yangu Pan, Zhaojun Teng, Minmin Gu, Chun Chen, Daniel Shek

**Affiliations:** ^1^Research Institute of Social Development, Southwestern University of Finance and Economics, Chengdu, China; ^2^Faculty of Psychology, Southwest University, Chongqing, China; ^3^School of Humanities and Social Science, The Chinese University of Hong Kong, Shenzhen, China; ^4^Department of Applied Social Sciences, The Hong Kong Polytechnic University, Kowloon, Hong Kong SAR, China

**Keywords:** social media use, internet use, smartphone use, problematic internet use, children and adolescents

## Introduction

Internet use (e.g., smartphone use, social media use) is almost indispensable in our daily lives and it plays an increasingly important role in shaping human wellbeing. Contemporary adolescents spend a significant amount of time on technological devices such as smartphones especially during COVID-19. As there is a strong need to understand the relationship between problematic Internet use [i.e., the incapacity to regulate one's Internet use, (1)] and psychological wellbeing of children and adolescents as well as the determinants of problematic Internet use, we launched this Research Topic project examining the association between problematic Internet use and psychological as well as behavioral outcomes. We also examined the related risk and protective factors in problematic Internet use.

There are several unique features of this Research Topic. First, this Research Topic investigates problematic Internet use among children and adolescents in different parts of the globe, including mainland China, Hong Kong, South Korea, Saudi Arabia, and Chile. This is important because existing studies in this field are dominated by “WEIRD” studies, with data collected predominantly from Western, educated, industrialized, rich, and democratic societies. The Research Topic comprises nine papers, with contribution from 43 investigators from mainland China, Hong Kong, South Korea, Saudi Arabia, UK, US, and Chile. Second, in response to the methodological issues highlighted by Shek (2), researchers in these studies employed a wide range of research designs including cross-sectional, longitudinal, experimental, and meta-analytic studies as well as secondary analyses of national data sets, with data collected before and after the COVID-19 pandemic. Third, this Research Topic highlights the risk and protective factors for problematic Internet use, such as coping and cyberbullying.

## Problematic Internet use and developmental outcomes

The studies in this Research Topic showed that there was a positive relationship between problematic Internet use and psychological and behavioral outcomes among children and adolescents, such as an increase in depressive and anxiety symptoms, physical and psychosocial symptoms, runaway behavior and aggression but a decrease in physical activity and life satisfaction.

Zhao et al. employed a longitudinal study to examine the association between Internet addiction and depressive and anxiety symptoms among Chinese adolescents. Chinese adolescents (*N* = 7,958) completed two-wave surveys before and during the COVID-19 pandemic (six-month interval). Results showed that psychological problems and Internet addiction significantly influenced each other.

Tsang et al. investigated the associations between electronic device use and the prevalence and severity of musculoskeletal symptoms, visual symptoms, psychosocial health, and quality of life in 1,058 primary and secondary school students in Hong Kong. Results revealed that excessive electronic device use was associated with increased prevalence and severity of physical and psychosocial symptoms, and such use was more prevalent in adolescents as compared to young children.

To examine the factors influencing adolescents' runaway behavior, Kim and Moon employed the national data of 11,354 adolescents from the Survey of Media Usage and Harmful Environment among adolescents in South Korea. They reported that exposure to harmful social media (e.g., adult online games, gambling games involving betting money or cyber money, messengers or chat apps for conditional dating) was an important antecedent of adolescent runaway behavior.

Al-Amri et al. used experimental and survey methods to examine the effects of smartphone addiction on the cognitive function and physical activity in middle-school children (*N* =196) recruited from middle schools in Saudi Arabia. Results showed some odd findings. While smartphone addiction was associated with a lower level of physical activity, it was related to better (i.e., not poorer) cognitive functioning.

Varela et al. investigated the moderating effect of various coping mechanisms on the association between social media addiction and adolescent life satisfaction during the COVID-19 pandemic period. Participants were 1,290 secondary school students in Chile. Results indicated that social media addiction and use of maladaptive stress coping strategies were risk factors associated with decreased life satisfaction among Chilean adolescents.

Li et al. conducted a meta-analytic study to examine the relationship between Internet gaming disorder and aggression among teenagers and young adults. They found that there was a significant relationship between Internet gaming disorder and aggression.

The studies in this Research Topic also identified some risk and protective factors for problematic Internet use. Risk factors included exposure to domestic violence, parental psychological control, childhood trauma, and online social anxiety. Besides, we identified several protective factors, including positive social control and self-control, parental behavioral control, positive parent-child relationships, and emotional intelligence as protective factors.

For example, Quancai et al. investigated the role of social control and self-control in the association between exposure to domestic violence and adolescent Internet gaming addiction. Participants were 2,110 adolescents recruited from Liangshan Yi Autonomous Prefecture in Sichuan Province, China. Results suggest that adolescent exposure to domestic violence increases adolescents' addiction to Internet games, and indirectly influences it through decreasing adolescents' social control and self-control.

Zhu et al. also investigated whether parent-child relationship moderated the association between parental control and adolescent Internet addiction. They recruited 1,974 Chinese adolescents in Guizhou Province in mainland China. Results indicated the positive impact of parental behavioral control and the negative impact of psychological control on the development of adolescent Internet addiction. Furthermore, positive father-adolescent relationship strengthened the positive effect of paternal behavioral control and mitigated the negative effect of psychological control of both parents on Internet addiction.

Furthermore, Cao et al. investigated the association between childhood trauma and adolescent cyberbullying perpetration as well as the mediating role of emotional intelligence and online social anxiety. Based on the responses of 1,046 adolescents from four schools in Shandong Province, China, results showed that childhood trauma was positively associated with adolescent cyberbullying perpetration. Moreover, emotional intelligence and online social anxiety mediated the association between childhood trauma and cyberbullying perpetration.

There are several significant contributions of this Research Topic. First, in view of the lack of systematic studies in this area in Chinese adolescents, this is a constructive contribution to the literature (3). Second, the Research Topic provides support for the social ecological model on adolescent development [e.g., (4)]. In particular, the studies in this Research Topic highlight the importance of personal as well as family ecological factors. Third, in response to the methodological issues highlighted by Shek (2), the studies in this study adopted different research designs with the recruitment of large samples. Finally, the present findings can help design effective intervention programs to reduce problematic Internet use and mental health problems in children and adolescents. Consistent with previous studies (5–7), the studies in this Research Topic highlight the importance of promoting emotional intelligence and self-control as well as parenting qualities to reduce problematic Internet use in children and adolescents.

## Author contributions

YP: Writing – original draft, Writing – review & editing. ZT: Writing – original draft. MG: Writing – original draft. CC: Writing – original draft. DS: Writing – original draft, Writing – review & editing.
